# Predicting the DPP-IV Inhibitory Activity pIC_50_ Based on Their Physicochemical Properties

**DOI:** 10.1155/2013/798743

**Published:** 2013-06-20

**Authors:** Tianhong Gu, Xiaoyan Yang, Minjie Li, Milin Wu, Qiang Su, Wencong Lu, Yuhui Zhang

**Affiliations:** ^1^School of Materials Science and Engineering, Shanghai University, 149 Yan-Chang Road, Shanghai 200072, China; ^2^Department of Chemistry, College of Sciences, Shanghai University, 99 Shang-Da Road, Shanghai 200444, China; ^3^Department of Neurosurgery, Changhai Hospital, Second Military Medical University, 168 Chang-Hai Road, Shanghai 200433, China

## Abstract

The second development program developed in this work was introduced to obtain physicochemical properties of DPP-IV inhibitors. Based on the computation of molecular descriptors, a two-stage feature selection method called mRMR-BFS (minimum redundancy maximum relevance-backward feature selection) was adopted. Then, the support vector regression (SVR) was used in the establishment of the model to map DPP-IV inhibitors to their corresponding inhibitory activity possible. The squared correlation coefficient for the training set of LOOCV and the test set are 0.815 and 0.884, respectively. An online server for predicting inhibitory activity pIC_50_ of the DPP-IV inhibitors as described in this paper has been given in the introduction.

## 1. Introduction

The incretin hormones glucagon-like peptide-1 (GLP-1) and glucose-dependent insulinotropic polypeptide (GIP) are the endogenous peptides that stimulate glucose-dependent insulin secretion [1]. One of the important roles of dipeptidyl peptidase IV (DPP-IV) [2] is a rapid inactivation of the GLP-1 and GIP. Inhibition of DPP-4 increases the levels of endogenous intact circulating GLP-1 and GIP. Consequently, inhibitors of DPP-4 or gliptins have been recently regarded as a prospective approach for the treatment of type-2 diabetes mellitus. 

In recent years, multiple small-molecule DPP-4 inhibitors have been reported [3, 4]. The development of a structurally diverse collection of DPP-4 inhibitors is a hot research [5–8]. Computational and various mathematical approaches have been widely employed in the quantitative structure-activity relationship (QSAR) analysis [9–13]. Using statistical methods, QSAR analyses were carried out on a dataset of 47 pyrrolidine analogs acting as DPP-IV inhibitors by Paliwal et al. [14]. Murugesan et al. used the comparative molecular field analysis (CoMFA) and comparative molecular similarity indices analysis (CoMSIA) to analyze the structural requirements of a DPP-IV active site [15]. Gao et al. developed a novel 3D-QSAR model to assist rational design of novel, potent, and selective pyrrolopyrimidine DPP-4 inhibitors [16]. Moreover, several efforts by using computational and mathematical approaches have been made in investigating small molecules of DPP-4 inhibitors. In our previous studies [17], we have attempted to use the quantum chemistry method [18] to optimize a series of DPP-IV inhibitors, and a 2D-QSAR model has been built, which can predict the inhibitory activity of small molecule with satisfying results. However, it is time consuming to calculate the molecular descriptors adopted in 2D-QSAR model.

In view of this, here we will try to devise an effective method to correctly recognize the possible activity prediction of small molecules based on physical and chemical properties of the compounds.

According to the general development trend [19, 20] and the recent research progress [21–31], the following procedures should be considered to establish a powerful statistical predictor for a biological system: (i) a valid benchmark dataset is constructed or selected to train and test the predictor; (ii) the samples are formulated with potent mathematical functions that are contributed to the prediction; (iii) a powerful algorithm is introduced or developed to operate the prediction; (iv) cross-validation tests are used to estimate the performance of the predictor; (v) a user-friendly online-server is established for the predictor that is accessible to the public. In this study, we attempt to describe how to deal with these steps for predicting the DPP-IV inhibitory activity pIC_50_ based on their physicochemical properties available via our program.

## 2. Materials and Methods

### 2.1. Data Preparation

**Figure 1:**
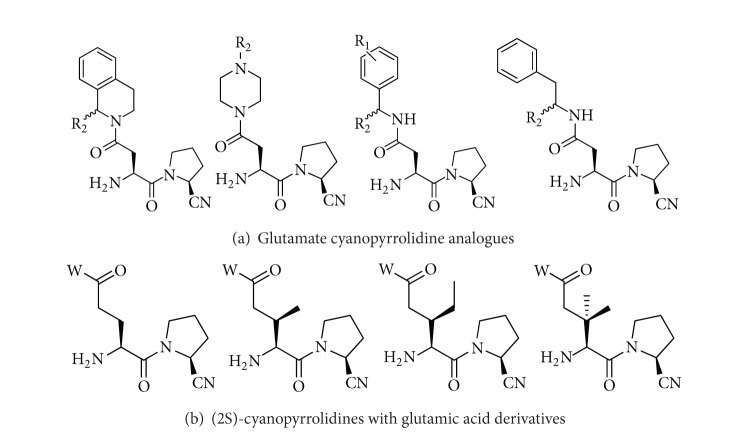
Molecular structure of cyanopyrrolidine amides as DPP-IV inhibitors.

The dataset used in the present work contains 48 pyrrolidine amides derivatives. In the current study, a diverse series of DPP-IV inhibitors with known IC_50_ values were collected from the papers [32, 33]. The detailed structures are documented in Supplementary Materials.(See Supplementary Material available at http://dx.doi.org/10.1155/2013/798743.) Figure 1 demonstrates the common structure of all of these analogues. All of the structures of compounds under investigation are based on the structure of Figure 1.

How to describe the molecules is an important problem in the establishment of the statistical model. In this study, the molecular descriptors for the 48 molecules were calculated by the second development software based on the calculator plugins, which is a product of ChemAxon [34]. ChemAxon is a company that provides chemical software development platforms and desktop applications for the biotechnology and pharmaceutical industries [35].

### 2.2. The Introduction of Procedure

Due to the use of Marvin Sketch graphic interface and JChem for Excel program, the calculations of small molecular descriptors are not very convenient. ChemAxon provides the calculation plugins of invoking function API, so our lab members have made a careful study and repeated experiments. The calculation results are compared with the ones of Gaussian 09 [18], JChem for Excel [34], HyperChem 7.5 [20, 36], and Dragon [37] programs calculation. By invoking the Calculator Plugins and using the Java language, we successfully developed a convenient and available customized batch calculation program (second development software) for the small molecular descriptors. 

**Figure 2:**
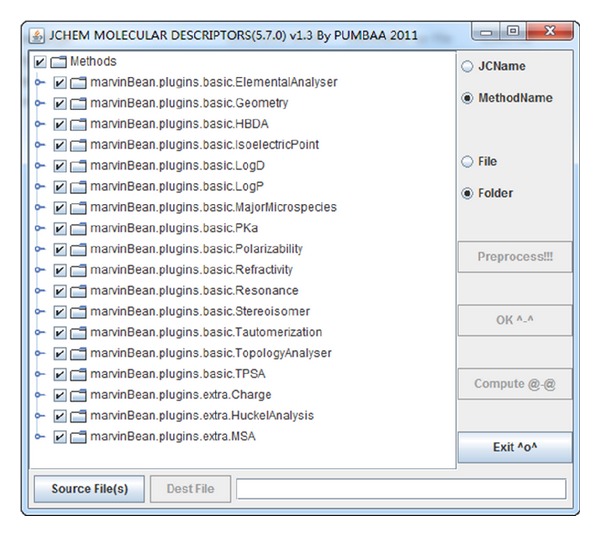
The program interface for the computation of molecular descriptors.

This program contains a selection of tree box; the user can choose the visual way to the calculation of molecular descriptors (as shown in Figure 2, command-line version does not provide molecular descriptor selection). The molecule structures are constructed from Gauss View 5.0 package [38, 39] as MOL-format file. Command-line version of the program is operated commonly in Linux server, through the similar execution command as follows:

*java-jar JChemCmd.jar Molecules Pathway Result.csv Method.xml *

### 2.3. Model Validation

#### 2.3.1. Dataset

The full dataset included training set (36 compounds) and test set (12 compounds). The whole samples were ranked by activity and were extracted every fourth sample for the generation of the test set.

#### 2.3.2. Leave-One-Out Cross-Validation (LOOCV) and Predictive Validation

In this study, Leave-one-out cross-validation (LOOCV) [40, 41] was used to investigate the prediction quality of training set. In the cross-validation, each sample is used to test the model that is established by all of the other samples at the same time.

#### 2.3.3. Fitting and Predictive Performances of Models

The fitting and predictive performances of model were measured by the squared correlation coefficient (*q* ^2^) and root mean square error (RMSE) for both the training set and the external test set. Here the performances of models can be estimated by *q* ^2^ and RMSE defined as follows, respectively:

(1) $$\begin{array}{c} q^{2}=1-\frac{\sum {\left(y_{i}-{\hat{y}}_{i}\right)}^{2}}{\sum {\left(y_{i}-y_{\text{mean}}\right)}^{2}},\\ \text{RMSE}=\sqrt{\frac{\sum {\left(y_{i}-{\hat{y}}_{i}\right)}^{2}}{N}}, \end{array}$$

where *y*_*i*_ and ${\hat{y}}_{i}$ are the actual and predicted pIC_50_ values of *i* sample, respectively, and *y*_mean_ is the average pIC_50_ value of the entire samples. *N* is the numbers of the training set.

### 2.4. Methods

For the sake of the redundancy of some features, the selection of descriptors before establishing a suitable model is necessary. The selection of descriptors plays an important role in construction for the actual model. In this work, mRMR-BFS method (minimum redundancy maximum relevance-backward feature selection) [42, 43] was used for the selection of molecular descriptors. The support vector regression (SVR) model was established based on the feature selection results. 

#### 2.4.1. mRMR-BFS Algorithm

The mRMR (minimum-redundancy maximum-relevance) algorithm was introduced by Ding and Ping [44], which was used usually for feature selection. It sorts a feature based on score function which is maximum relevance to target and minimum redundancy to the already selected features. The score function is defined as follows:

(2) $$\begin{array}{c} \text{scor}{\text{e}}_{j}=I\left(f_{j},c\right)-\frac{1}{m}\sum_{i=1}^{m}I\left(f_{i},f_{j}\right), \end{array}$$

where *f*_*j*_ ⊂ *S*_*n*_, *f*_*i*_ ⊂ *S*_*m*_, *S*_*m*_ = *S* − *S*_*n*_,  and *S*_*m*_, *S*_*n*_, and *S* are the feature sets. *m* and *n* are the feature numbers. The mutual information *I*(*x*, *y*) is as follows:

(3) $$\begin{array}{c} I\left(x,y\right)=\iint p\left(x,y\right)\log\frac{p\left(x,y\right)}{p\left(x\right)p\left(y\right)}dx\;dy, \end{array}$$

where *p*(*x*, *y*), *p*(*x*), and *p*(*y*) are the probabilistic density functions.

More details about mRMR algorithm can be found in [44, 45].

To gain an even better performance of predictor and feature selection, backward feature selection (BFS) based on the result of mRMR is also used in this study. The most important 50 variables were obtained from the mRMR procedure. We initialize the BFS-selected feature set *S*_*s*_ with all features in *S*:

(4) $$\begin{array}{c} S_{s}=\left\{f_{1}^{\prime },f_{2}^{\prime },\ldots ,f_{k}^{\prime }\right\}\;\left(1\leq k\leq 50\right). \end{array}$$

With the mRMR-selected feature subset *S*_*s*_, the next BFS-selected feature set can be gained by the following steps.

1. Suppose that the candidate feature set is *S*_*C*_ = *S*_*S*_ − *f*_*k*_. Then an SVR model based on each *S*_*C*_ is established and evaluated by LOOCV method.
2. The feature *f* which gets the lowest RMSE is selected when removed from *S*_*S*_.
3. The feature *f* is removed from *S*_*s*_ forming the next BFS-selected feature set.

#### 2.4.2. SVM (Support Vector Machine)

Vapnik and his co-workers developed the SVM algorithm, which is a supervised machine-learning method that is used for classification and regression analysis. Owing to embodying the structural risk minimization principle, the SVM exhibits a better whole performance. The SVM is suitable for the problems which are involved in the small sample set. In this work, SVM was applied to regression. The details of the algorithm can be found in reference [46]. The algorithm was performed by using the software package Weka 3.6.7 [47, 48].

## 3. Results and Discussion

### 3.1. Selection of Features

Firstly, mRMR method was applied to rank the total 75 features according to their mRMR scores. Secondly, we used the backward feature selection (BFS) algorithm based on SVR to search for the feature combinations. As different machine learning methods will lead to different results, several robust machine learning methods like the nearest-neighbor algorithm (NNA), support vector machine (SVM based on RBF kernel function), and Adaboost were employed to find an optimal feature subset with leave-one-out cross-validation, respectively. As a result, we adopted the SVM as the prediction engine based on the LOOCV in this study.

Table 1 lists an optimal subset attained by employing the above two-stage feature selection method, mRMR-BFS. The six features in optimal subset can be clustered into three categories (based on the category of Calculator Plugins [49]): elemental analysis, geometry, topology, and others. The geometry and topology factor are more important in this work. The geometry and topology factor are related to the size of the molecule as it indicates that the size of cyanopyrrolidine amides derivatives plays a main role in the inhibitory activity.

### 3.2. Results of Computation

In this work, *q*_train_^2^, *q*_train-CV_^2^, and *q*_test_^2^ were used to present the squared correlation coefficients for the training set, cross-validation set, and external test set, respectively. Also RMSE_train_, RMSE_train-CV_, and RMSE_test_ were adopted to present the root mean square errors for the training set, cross-validation set, and external test set, respectively. 

The final model was built by the SVR based on the Gaussian kernel function (RBF) with the parameters  *C*, *ε*, and *γ* that are 2.0, 0.05, and 1.0, respectively. The Gaussian kernel function (RBF) is given as follows:

(5) $$\begin{array}{c} K\left(x,x_{i}\right)=\exp\left(-\gamma {||x-x_{i}||}^{2}\right). \end{array}$$

The model based on the above parameters with original data is given as follows:

(6) $$\begin{array}{c} \text{pI}{\text{C}}_{50}=2.10*\left[\sum_{i\subset \text{SV}}\beta_{i}\exp\left(-{||x-x_{i}||}^{2}\right)+0.207\right]+6.60, \end{array}$$

where *β*_*i*_ is the Lagrange coefficient of support vectors.

**Figure 3:**
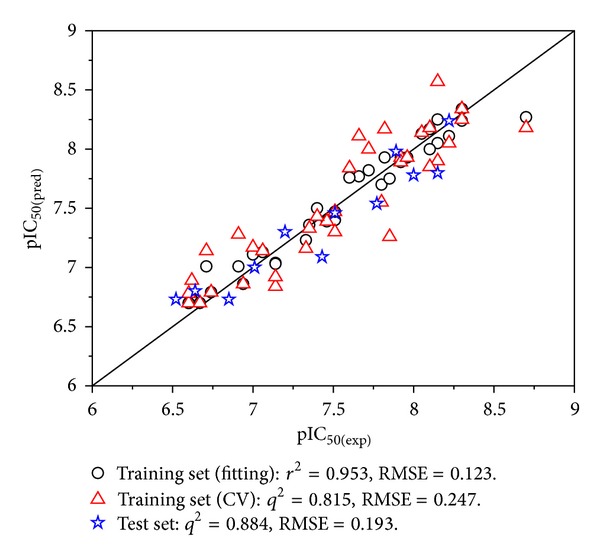
Predicted versus experimental pIC_50_ for the training (circles for fitting and triangle for CV, respectively) and test (stars) sets.

**Figure 4:**
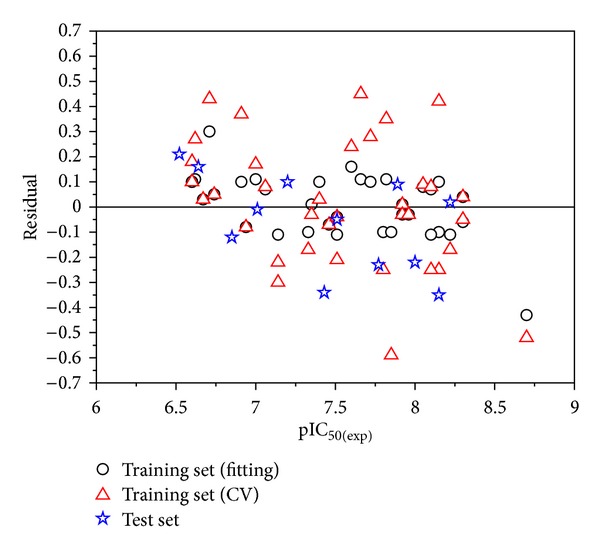
Dispersion plot of the residuals for the training and test sets.

The experimental versus predicted pIC_50_ values based on the SVR model for the training set and test set are shown in Figure 3. As a result, the values of *q*_train_^2^, *q*_train-CV_^2^, and *q*_test_^2^ were 0.953, 0.815, and 0.884, respectively. And the values of RMSE_train_, RMSE_train-CV_, and RMSE_test_ were 0.123, 0.247, and 0.193, respectively. Figure 3 illustrates that the regression straight line is appropriate not only for the fitting pIC_50_ values of the training set but also for the predicted pIC_50_ values of the external test set.  Table 2 shows the experimental and the calculated values over the training set and the test set. From Figure 3 and Table 2, it can be concluded that the predicted values are in good agreement with the experimental ones.  Figure 4 illustrates the dispersion plot of the residuals for the training and test sets. The predicted values are randomly dispersed around the zero-value line in Figure 4. It means that the model is appropriate for the data.

### 3.3. Analysis of the New Method

The secondary development program developed in this work was used to establish a robust model with *q*_train_^2^ = 0.953, *q*_train-CV_^2^ = 0.815, and *q*_test_^2^ = 0.884,  respectively. In order to validate the generalization and reliability of the descriptors obtained by using our secondary development program, the same training and test sets were also constructed and optimized at the HF/6 − 31G* level of theory with the Gaussian program; 1262 descriptors were computed by HyperChem 7.5 program [20], JChem for Excel package [34], and the Dragon program [37]. And a robust and reliable model was obtained with *q*_train_^2^ = 0.969, *q*_train-CV_^2^ = 0.868, and *q*_test_^2^ = 0.891,  respectively. The statistical comparisons were summarized in Table 3.

It is indicated that it takes less than 30 minutes for a molecule from the structure optimization to the computation of descriptors by using the second development program. In contrast, more than 36 hours were taken based on the Gaussian program. These results show that the computing speeds are greatly improved by using the secondary development program, while the statistical parameters of models are as good as those obtained with the Gaussian method. Therefore, the second development program is very helpful not only for saving the time of descriptor computation but also for providing the effective QSPR models online available in the future.

 In a benchmark test, the support vector regression (SVR) was contrasted with the multiple linear regression (MLR) and the back propagation-artificial neural network (BP-ANN) on the *q*_train-CV_^2^. The statistical comparisons were shown in Table 4. From Table 4, SVR has a better generalization ability in our work.

### 3.4. The Online Web Server

Since user-friendly and publicly accessible online servers represent the trend for developing more useful models or predictors, we established a web server for predicting the DPP-IV inhibitory activity pIC_50_ at http://chemdata.shu.edu.cn:8080/QSARPrediction/index.jsp.

The web server allows users to upload the MOL-format file of a molecule, and the server will return the result of prediction according to the model of our mRMR-BFS-SVR method. In this course, the Calculator Plugins [49] of ChemAxon was invoked in the background program. The server developed has the most outstanding characteristic that users need to do nothing except for uploading the file of the unknown small molecule. Then they can get the predicted result after waiting for some time. It is a remarkable advance compared to our previous work [17, 20, 36].

## 4. Conclusions 

In this paper, the secondary development program was proposed to bring an efficient and fast calculation means for molecular descriptors. The mRMR-BFS was adopted in the procedure of feature selection. The SVR was used to construct the model to map DPP-IV inhibitors to their corresponding inhibitory activity. The *q*_train_^2^, *q*_train-CV_^2^, and *q*_test_^2^ of the model are 0.953, 0.815, and 0.884, respectively. These results are as good as those obtained with the Gaussian method. The web server, which provides a quick approach to predict the DPP-IV inhibitory activities pIC_50_ of unknown small molecules based on their MOL-format files, was established by using our secondary development program at http://chemdata.shu.edu.cn:8080/QSARPrediction/index.jsp. A user-friendly and rapid approach whose accuracy is approximate with the Gaussian method is proposed in this work.

## Supplementary Material

A full list of the structure and molecular descriptors of compound are available in the supplementary Materials.

## Figures and Tables

**Table 1:** Symbols for molecular descriptors involved in the model.

Molecular descriptor	Type	Description
OComposition	Elemental analysis functions	O Composition
MaximalProjectionArea	Geometry	Calculates the maximal projection area
MinimalProjectionArea	Geometry	Calculates the minimal projection area
BasicpKa	pKa	Constant denoting basic pKa
RingBondCount	Topology	Ring bond count
AliphaticRingCount	Topology	Aliphatic ring count

**Table 2:** Experimental and predicted pIC_50_ for the training and test sets.

No.	pIC_50(exp)_	pIC_50(Pred)_	pIC_50(LOOCV)_
1	7.00	7.11	7.17
2^T^	7.20	7.30	—
3	7.35	7.36	7.33
4	7.33	7.23	7.16
5^T^	7.01	7.00	—
6	7.14	7.04	6.92
7	7.14	7.03	6.84
8	6.71	7.01	7.14
9^T^	6.64	6.80	—
10	7.06	7.13	7.14
11	6.91	7.01	7.28
12	6.62	6.73	6.89
13	6.60	6.70	6.78
14^T^	6.85	6.73	—
15	6.67	6.70	6.70
16	6.60	6.70	6.70
17	6.94	6.86	6.86
18	6.74	6.79	6.79
19^T^	6.52	6.73	—
20	8.70	8.27	8.18
21	8.30	8.34	8.34
22	7.46	7.39	7.39
23	7.40	7.50	7.43
24^T^	8.22	8.24	—
25	8.15	8.25	8.57
26	8.30	8.24	8.25
27	8.05	8.13	8.14
28	8.22	8.11	8.05
29	8.15	8.05	7.90
30^T^	8.00	7.78	—
31	7.66	7.77	8.11
32^T^	8.15	7.80	—
33	7.82	7.93	8.17
34^T^	7.77	7.54	—
35^T^	7.51	7.46	—
36	8.10	8.00	7.85
37	7.72	7.82	8.00
38^T^	7.43	7.09	—
39	7.96	7.93	7.93
40	8.10	8.17	8.18
41	7.51	7.40	7.30
42	7.92	7.89	7.89
43	7.51	7.47	7.47
44	7.92	7.93	7.93
45	7.80	7.70	7.55
46	7.60	7.76	7.84
47	7.85	7.75	7.26
48^T^	7.89	7.98	—

^T^indicates the test samples.

**Table 3:** Comparative statistical parameters obtained by the secondary development program and the Gaussian program concerning the same compounds.

Program	*q*_train_^2^	*q*_train-CV_^2^	*q*_test_^2^
The secondary development program developed in this work	0.953	0.815	0.884

Gaussian, HyperChem 7.5, JChem for Excel package, Dragon	0.969	0.868	0.891

**Table 4:** *q*_train-CV_^2^ of different methods.

Method	SVR	BP-ANN	MLR
*q*_train-CV_^2^	0.815	0.761	0.721
