# Supplementary material for: Predicting the DPP-IV Inhibitory Activity pIC50 Based on Their Physicochemical Properties
Source: Biomed Res Int. 2013 Jun 20;2013:798743. doi: 10.1155/2013/798743 (PMC3705804; doi:10.1155/2013/798743)
Supplement: Supplementary file 1 — A full list of the structure and molecular descriptors of compound are available in the supplementary Materials. [file 798743.f1.pdf]

Supplementary Material

Table Structural classes of compounds with pIC_{50} of pyrrolidine based DPP-IV inhibitors used in the QSAR analysis

A) Glutamate cyanopyrrolidine analogues

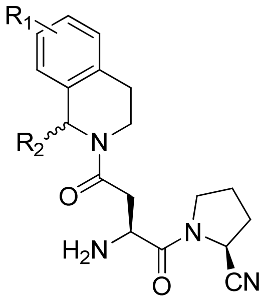

1-8

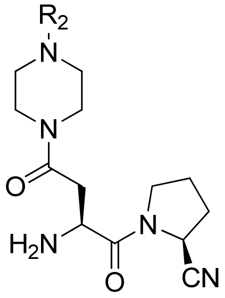

9-11

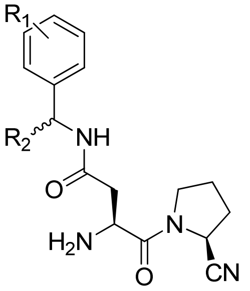

12-16

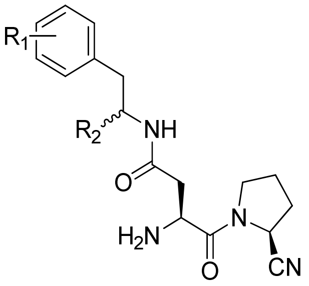

16-19

Comp	R_1	R_2
1	H	H
2	6,7-$(OMe)_2$	H
3	6,7-$(OMe)_2$	-$(CH_2)_2OH$
4	6,7-$(OMe)_2$	Isopropyl
5	6,7-$(OMe)_2$	Benzyl
6	6,7-$(OMe)_2$	tert-Butyl
7	6-OMe	tert-Butyl
8	7-OMe	tert-Butyl
9	H	$CH(4\text{-}FC_6H_5)_2$
10	H	Nicotinonitrile
11	H	Benzoyl
12	H	H
13	H	Benzyl
14	H	Ethyl
15	H	Isopropyl
16	H	tert-Butyl

17	3,4-OMe	H
18	H	CH_2OMe
19	H	Isopropyl

Compounds 1 to 19 taken from reference 23

(B) (2S)-cyanopyrrolidines with glutamic acid derivatives

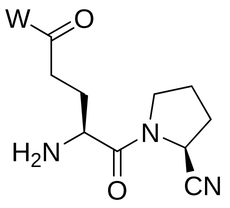

20-39

Comp	W
20	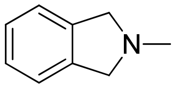
21	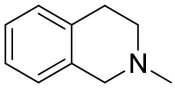
22	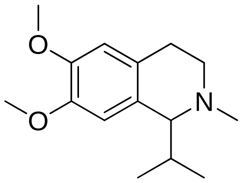
23	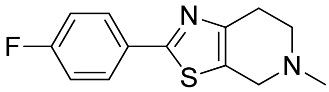
24	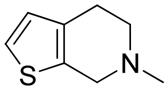
25	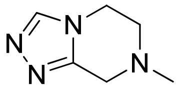
26	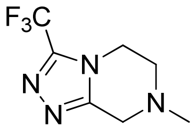
27	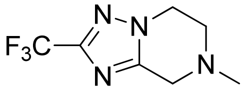

28	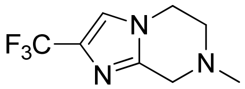
29	1-pyrrolidine
30	1-thiazolidine
31	1-(R)-2-(methoxymethyl)pyrrolidine
32	1-(S)-2-(methoxymethyl)pyrrolidine
33	1-piperidine
234	1-morpholine
35	1- piperazine-4- (ethyl) carboxylate
36	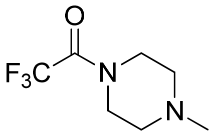
37	1-(pyridin-4-yl)piperazine
38	N-aniline
39	N-cyclopentanamine

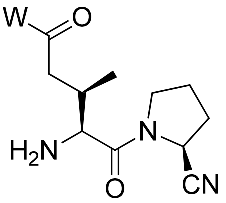

40

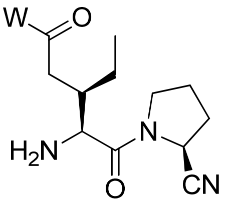

41

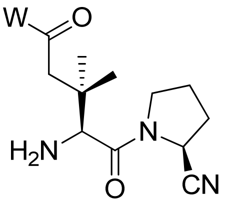

41-48

Comp	W
40	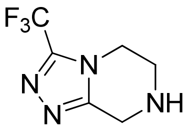
41	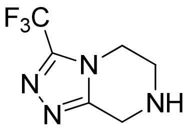
42	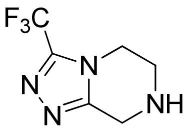

43	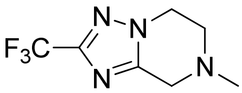
44	N-isoindoline
45	N-tetrahydroisoquinoline
46	N-tetrahydrothieno[2,3-c]pyridine
47	N-(R)-2-methoxypyrrolidine
48	N-(R)-2-methylpyrrolidine

Compounds 20 to 48 taken from reference 24

All the features data are shown in following table.

No	pIC50	AllAtom Count	Mass	ExactMass	CCompo sition	HCompo sition	OCompo sition	NComposition	SComposition	XComposition	Dreiding Energy
1	7	46	326.3929	326.1743	66.24	6.79	9.8	17.17	0	0	67.78
2	7.2	54	386.4448	386.1954	62.16	6.78	16.56	14.5	0	0	78.75
3	7.35	61	430.4974	430.2216	61.38	7.02	18.58	13.01	0	0	92.48
4	7.33	63	428.5246	428.2424	64.46	7.53	14.93	13.07	0	0	106.2
5	7.01	67	476.5674	476.2424	68.05	6.77	13.43	11.76	0	0	110.85
6	7.14	66	442.5512	442.258	65.14	7.74	14.46	12.66	0	0	100.9
7	7.14	62	412.5252	412.2474	66.96	7.82	11.64	13.58	0	0	104.81
8	6.71	62	412.5252	412.2474	66.96	7.82	11.64	13.58	0	0	104.34
9	6.64	64	515.983	515.19	60.52	5.47	6.2	13.57	0	14.23	108.65
10	7.06	51	381.4316	381.1913	59.83	6.08	8.39	25.7	0	0	84.86
11	6.91	53	383.4442	383.1957	62.65	6.57	12.52	18.26	0	0	86.74
12	6.62	42	300.3556	300.1586	63.98	6.71	10.65	18.65	0	0	47.69
13	6.6	55	390.4781	390.2056	70.75	6.71	8.19	14.35	0	0	66.24
14	6.85	48	328.4088	328.1899	65.83	7.37	9.74	17.06	0	0	53.45
15	6.67	51	342.4353	342.2056	66.64	7.65	9.34	16.36	0	0	58.5
16	6.6	54	356.4619	356.2212	67.39	7.92	8.98	15.72	0	0	66.09
17	6.94	53	374.4341	374.1954	60.95	0	17.09	14.96	0	0	62.26
18	6.74	52	358.4347	358.2005	63.67	7.31	13.39	15.63	0	0	57.69
19	6.52	54	356.4619	356.2212	67.39	7.92	8.98	15.72	0	0	63.73
20	8.7	46	326.3929	326.1743	66.24	6.79	9.8	17.17	0	0	58.84
21	8.3	49	340.4195	340.1899	67.04	7.11	9.4	16.46	0	0	67.41
22	7.46	66	442.5512	442.258	65.14	7.74	14.46	12.66	0	0	95.89
23	7.4	55	441.522	441.1635	59.85	5.48	7.25	15.86	7.26	4.3	89.78

24	8.22	46	346.447	346.1463	58.94	6.4	9.24	16.17	9.26	0	75.8
25	8.15	45	331.3729	331.1757	54.37	6.39	9.66	29.59	0	0	68.87
26	8.3	48	399.3709	399.1631	48.12	5.05	8.01	24.55	0	14.27	70.53
27	8.05	48	399.3709	399.1631	48.12	5.05	8.01	24.55	0	14.27	71.66
28	8.22	49	398.3828	398.1678	51.25	5.31	8.03	21.1	0	14.31	73.97
29	8.15	42	278.3501	278.1743	60.41	7.97	11.5	20.13	0	0	46.95
30	8	40	296.388	296.1307	52.68	6.8	10.8	18.9	10.82	0	55.15
31	7.66	49	322.4026	322.2005	59.61	8.13	14.89	17.38	0	0	53.18
32	8.15	49	322.4026	322.2005	59.61	8.13	14.89	17.38	0	0	57.76
33	7.82	45	292.3767	292.1899	61.62	8.27	10.94	19.16	0	0	56.74
34	7.77	43	294.3495	294.1692	57.13	7.53	16.31	19.03	0	0	56.67
35	7.51	53	365.4274	365.2063	55.87	7.45	17.51	19.16	0	0	65.04
36	8.1	49	389.3728	389.1675	49.35	5.69	12.33	17.99	0	14.64	67.13
37	7.72	53	370.4487	370.2117	61.6	7.07	8.64	22.69	0	0	87.78
38	7.43	42	300.3556	300.1586	63.98	6.71	10.65	18.65	0	0	53.1
39	7.96	45	292.3767	292.1899	61.62	8.27	10.94	19.16	0	0	49.15
40	8.1	51	413.3975	413.1787	49.39	5.36	7.74	23.72	0	13.79	76.09
41	7.51	54	427.4241	427.1944	50.58	5.66	7.49	22.94	0	13.33	78.89
42	7.92	54	427.4241	427.1944	50.58	5.66	7.49	22.94	0	13.33	90.28
43	7.51	54	427.4241	427.1944	50.58	5.66	7.49	22.94	0	13.33	87.64
44	7.92	52	354.446	354.2056	67.77	7.39	9.03	15.81	0	0	77.83
45	7.8	55	368.4726	368.2212	68.45	7.66	8.68	15.21	0	0	89.9
46	7.6	52	374.5	374.1776	60.94	0	8.54	14.96	8.56	0	92.69
47	7.85	55	350.4558	350.2318	61.69	8.63	13.7	15.99	0	0	71.62
48	7.89	51	320.4298	320.2212	63.72	8.81	9.99	17.48	0	0	65.75

No	MaximalProjectionArea	MaximalProjectionRadius	MinimalProjectionArea	MinimalProjectionRadius	Volume	MaxZ	MinZ	AcceptorCount	DonorCount	AcceptorSiteCount
1	90.21782	8.050012	50.51889	4.485333	303.857	8.562943	16.01556	4	1	6
2	105.4927	9.163982	57.28759	5.114214	355.9446	8.618475	17.96585	6	1	8
3	122.4488	9.510876	52.76911	5.318754	399.3231	8.248095	19.05898	7	2	9
4	113.3451	9.618911	54.95064	4.850853	407.7508	9.182845	19.31841	6	1	8
5	131.9493	9.501111	74.23499	6.320266	444.4602	7.957241	18.35312	6	1	8
6	122.1118	9.271526	60.14329	5.27785	424.8856	9.149217	18.53774	6	1	8
7	111.8175	9.590354	53.85912	4.753837	398.8984	8.73516	18.45883	5	1	7
8	114.7854	9.435717	53.92403	4.827021	399.0399	8.690177	18.68843	5	1	7
9	115.7996	9.886934	72.12349	6.046644	446.5367	9.419478	19.26047	5	1	7
10	107.5889	10.3868	45.84579	4.110203	345.9536	8.042938	20.78185	7	1	9
11	98.54881	8.518206	60.07124	5.299663	352.2171	9.138657	16.92138	5	1	8
12	86.43198	8.155561	43.84907	4.088075	280.1381	9.555555	16.1839	4	2	6
13	107.1037	7.929799	68.10867	6.315899	368.0984	9.489547	16.37244	4	2	6
14	90.94059	8.092101	53.61216	5.120422	314.4529	9.840346	15.54838	4	2	6
15	95.70338	7.934439	52.95323	4.939686	331.6427	8.998769	15.82861	4	2	6
16	96.97322	7.957213	56.8263	4.941445	348.8861	10.13762	15.85103	4	2	6
17	100.2765	8.920777	48.99909	4.62372	349.2715	7.97274	17.85642	6	2	8
18	98.19989	8.061612	56.43587	5.917173	340.5827	9.546245	16.1321	5	2	7
19	94.09718	7.830166	59.55977	5.100545	348.5061	8.971077	15.62451	4	2	6
20	96.65496	8.414302	47.90958	4.672555	302.8909	6.681803	16.75393	4	1	6
21	103.2934	8.412557	45.73021	4.993703	320.8645	7.245613	15.82804	4	1	6
22	119.7228	9.67974	66.58052	5.910181	424.2845	10.45237	19.08871	6	1	8
23	127.4939	10.27434	57.59772	5.585624	383.2633	6.691685	20.00208	5	1	7

24	101.1197	8.222265	51.00596	4.818358	312.1294	6.504986	16.32724	4	1	6
25	95.18543	8.088551	47.95331	4.524636	296.5216	6.877353	15.70946	6	1	8
26	103.0209	8.829092	49.49292	4.530797	328.0844	7.069434	17.14262	6	1	8
27	103.8436	8.633797	53.00252	5.578315	327.8928	6.802793	17.27336	6	1	8
28	105.5932	8.63286	53.36846	5.583361	332.3927	6.809747	17.27242	5	1	7
29	84.45585	7.072092	46.80155	4.65293	266.3014	6.791361	13.98269	4	1	6
30	87.86735	7.574191	40.64974	4.294963	269.0596	6.9876	15.16345	4	1	6
31	96.96373	7.491767	52.69191	5.038903	309.7007	7.182907	14.74647	5	1	7
32	93.94895	7.982574	54.13586	5.331433	309.939	8.602409	15.88809	5	1	7
33	90.86448	7.676783	42.47848	4.482025	284.1974	7.144694	15.02331	4	1	6
34	89.2268	7.661667	42.49947	4.685857	276.4744	7.209152	15.36368	5	1	7
35	110.5458	9.116353	51.95143	5.778303	342.0249	8.50987	16.93555	5	1	8
36	103.4188	8.754236	48.68843	5.012881	330.9904	7.086668	17.40452	5	1	8
37	108.4972	9.042902	58.0398	5.156832	346.3938	8.021161	17.95568	6	1	8
38	85.95381	8.423172	41.14845	4.079129	280.3215	7.448482	16.61165	4	2	6
39	84.50367	8.061011	43.56521	4.24572	283.1348	7.149977	16.09849	4	2	6
40	108.1727	8.809023	52.59644	5.234514	345.5271	6.807088	17.35802	6	1	8
41	103.8295	7.963187	66.85119	6.427705	362.4276	9.998837	15.68488	6	1	8
42	104.2344	9.085406	52.44393	4.824977	362.8101	8.94882	18.19945	6	1	8
43	104.3395	8.632966	61.22184	5.572285	362.6896	9.105379	17.32514	6	1	8
44	100.5154	8.414709	53.42224	5.648362	337.821	7.85431	16.46374	4	1	6
45	109.9819	8.46209	57.38174	5.581879	355.895	7.972607	16.855	4	1	6
46	106.671	8.087825	56.21323	5.509178	346.9997	8.434003	16.29725	4	1	6
47	84.08339	6.15481	67.68598	5.536571	344.73	10.31215	10.93144	5	1	7
48	78.57374	5.790844	61.53946	5.385834	318.2618	9.574804	10.01881	4	1	6

No	DonorSite Count	LogD	LogP	Microspecies Count	AcidicpKa	BasicpKa	LargeModel AcidicpKa	LargeModel BasicpKa	Polarizability	Refractivity
1	2	-0.29222	-0.02166	16	19.37356	7.337513	19.37356	7.337513	34.75897	90.0363
2	2	-0.60756	-0.337	64	19.37356	7.337513	19.37356	7.337513	39.83058	102.9627
3	3	-1.17793	-0.90737	128	15.91124	7.337507	15.91124	7.337507	44.14995	113.7906
4	2	0.696513	0.967071	64	19.37181	7.337507	19.37181	7.337507	45.33546	116.3771
5	2	1.465344	1.735902	64	19.37178	7.337507	19.37178	7.337507	51.22832	132.0005
6	2	1.074514	1.345072	64	19.3717	7.337507	19.3717	7.337507	47.17215	120.7752
7	2	1.232185	1.502743	32	19.3717	7.337507	19.3717	7.337507	44.64052	114.312
8	2	1.232185	1.502743	32	19.3717	7.337507	19.3717	7.337507	44.64053	114.312
9	2	1.780714	2.062113	32	19.36084	7.349998	19.36084	7.349998	50.78548	131.8613
10	2	-1.08547	-0.8149	64	19.37479	7.337525	19.37479	7.337525	38.72608	102.5409
11	2	-1.12839	-0.85783	32	19.37479	7.337518	19.37479	7.337518	39.47253	103.1059
12	3	-0.5559	-0.28522	32	14.70965	7.33777	14.70965	7.33777	31.80921	81.83
13	3	1.517002	1.78768	32	14.7241	7.337765	14.7241	7.337765	43.23304	110.8678
14	3	0.383198	0.653876	32	14.70635	7.337765	14.70635	7.337765	35.48539	90.7728
15	3	0.748171	1.018849	32	14.74449	7.337765	14.74449	7.337765	37.32481	95.2444
16	3	1.126172	1.39685	32	14.77881	7.337765	14.77881	7.337765	39.16486	99.6425
17	3	-0.58259	-0.3119	128	14.64979	7.337776	14.64979	7.337776	38.71952	99.5114
18	3	-0.25445	0.016232	64	14.73997	7.33777	14.73997	7.33777	38.0339	97.2987
19	3	1.03683	1.30751	32	14.95677	7.33777	14.95677	7.33777	39.16739	99.9994
20	2	-0.89763	-0.02166	8	22.05959	8.216998	22.05959	8.216998	34.75693	90.0363
21	2	-0.60897	0.267005	8	22.0596	8.216998	22.0596	8.216998	36.5974	94.7913
22	2	0.379761	1.255732	32	22.05959	8.216998	22.05959	8.216998	47.17219	121.1321
23	2	0.37956	1.255532	32	22.0596	8.216998	22.0596	8.216998	44.36217	124.524

24	2	-0.69609	0.179886	16	22.0596	8.216998	22.0596	8.216998	35.29345	91.6812
25	2	-3.60246	-2.72649	32	22.0596	8.216998	22.0596	8.216998	32.63002	86.9074
26	2	-2.34707	-1.47109	32	22.0596	8.216998	22.0596	8.216998	33.95294	92.3583
27	2	-1.39712	-0.52026	32	22.0596	8.216998	22.0596	8.216998	33.94554	102.5518
28	2	-1.37834	-0.50234	16	22.0596	8.217003	22.0596	8.217003	34.73424	92.2929
29	2	-1.96753	-1.09156	8	22.0596	8.216998	22.0596	8.216998	28.88006	74.4101
30	2	-2.10713	-1.23116	8	22.05959	8.216998	22.05959	8.216998	30.11923	77.1699
31	2	-1.95474	-1.07877	16	22.0596	8.216998	22.0596	8.216998	33.27057	85.1238
32	2	-1.95474	-1.07877	16	22.0596	8.216998	22.0596	8.216998	33.27057	85.1238
33	2	-1.52296	-0.64699	8	22.0596	8.216998	22.0596	8.216998	30.71389	79.0111
34	2	-2.59183	-1.71586	16	22.0596	8.216998	22.0596	8.216998	29.60941	75.9436
35	2	-2.32322	-1.44724	8	22.0596	8.216998	22.0596	8.216998	36.37985	93.5626
36	2	-2.16699	-1.29101	8	22.0596	8.216998	22.0596	8.216998	33.31463	88.2006
37	2	-2.68738	-0.97679	32	22.0596	8.822567	22.0596	8.822567	38.70567	101.2608
38	3	-0.57943	0.296535	16	14.20096	8.216995	14.20096	8.216995	31.80063	83.5308
39	3	-1.24231	-0.36633	16	14.8862	8.217002	14.8862	8.217002	30.71389	78.3856
40	2	-2.03961	-1.10612	32	21.62027	8.283136	21.62027	8.283136	35.75493	96.8299
41	2	-1.58815	-0.66155	32	21.51441	8.275276	21.51441	8.275276	37.56013	101.4309
42	2	-1.4271	-0.72812	32	21.19529	8.004089	21.19529	8.004089	37.56013	101.228
43	2	-0.40473	0.294792	32	21.19529	8.004089	21.19529	8.004089	37.55263	111.4215
44	2	0.022337	0.721317	8	21.19529	8.004089	21.19529	8.004089	38.43451	98.906
45	2	0.310998	1.009978	8	21.19531	8.004089	21.19531	8.004089	40.27635	103.661
46	2	0.22388	0.92286	16	21.1953	8.004089	21.1953	8.004089	38.9609	100.5509
47	2	-1.03477	-0.33579	16	21.19531	8.004089	21.19531	8.004089	36.93728	93.9935
48	2	-0.63099	0.067989	8	21.19531	8.004089	21.19531	8.004089	34.38519	87.6986

No	Resonant Count	Stereo IsomerCount	TetrahedralStereo IsomerCount	Tautomer Count	Bond Count	Cyclomatic Number	Ring Count	RingAtom Count	RingBond Count	ChainAtom Count	ChainBond Count
1	18	4	4	20	48	3	3	15	16	9	10
2	18	4	4	20	56	3	3	15	16	13	14
3	18	8	8	20	63	3	3	15	16	16	17
4	18	8	8	20	65	3	3	15	16	16	17
5	18	8	8	20	70	4	4	21	22	14	16
6	18	8	8	20	68	3	3	15	16	17	18
7	18	8	8	20	64	3	3	15	16	15	16
8	18	8	8	20	64	3	3	15	16	15	16
9	18	8	8	20	67	4	4	23	23	13	16
10	450	4	4	20	53	3	3	17	17	11	13
11	54	4	4	20	55	3	3	17	17	11	13
12	18	4	4	28	43	2	2	11	11	11	12
13	18	8	8	28	57	3	3	17	17	12	14
14	18	8	8	28	49	2	2	11	11	13	14
15	18	8	8	28	52	2	2	11	11	14	15
16	18	8	8	28	55	2	2	11	11	15	16
17	18	4	4	28	54	2	2	11	11	16	17
18	18	8	8	28	53	2	2	11	11	15	16
19	18	8	8	28	55	2	2	11	11	15	16
20	18	4	4	32	48	3	3	14	15	10	11
21	18	4	4	32	51	3	3	15	16	10	11
22	18	8	8	32	68	3	3	15	16	17	18
23	144	4	4	32	58	4	4	20	21	11	13

24	126	4	4	32	48	3	3	14	15	10	11
25	162	4	4	32	47	3	3	14	15	10	11
26	162	4	4	32	50	3	3	14	15	14	15
27	180	4	4	32	50	3	3	14	15	14	15
28	144	4	4	32	51	3	3	14	15	14	15
29	18	4	4	32	43	2	2	10	10	10	11
30	18	4	4	32	41	2	2	10	10	10	11
31	18	8	8	32	50	2	2	10	10	13	14
32	18	8	8	32	50	2	2	10	10	13	14
33	18	4	4	32	46	2	2	11	11	10	11
34	18	4	4	32	44	2	2	11	11	10	11
35	72	4	4	32	54	2	2	11	11	15	16
36	54	4	4	32	50	2	2	11	11	16	17
37	108	4	4	32	55	3	3	17	17	10	12
38	18	4	4	42	43	2	2	11	11	11	12
39	18	4	4	42	46	2	2	10	10	11	12
40	162	8	8	40	53	3	3	14	15	15	16
41	162	8	8	48	56	3	3	14	15	16	17
42	162	4	4	12	56	3	3	14	15	16	17
43	180	4	4	12	56	3	3	14	15	16	17
44	18	4	4	12	54	3	3	14	15	12	13
45	18	4	4	12	57	3	3	15	16	12	13
46	126	4	4	12	54	3	3	14	15	12	13
47	18	8	8	12	56	2	2	10	10	15	16
48	18	8	8	12	52	2	2	10	10	13	14

No	Aliphatic RingCount	Aromatic RingCount	Aliphatic AtomCount	Aliphatic BondCount	Aromatic AtomCount	Aromatic BondCount	Carbo RingCount	Hetero RingCount	HeteroAromatic RingCount	CarboAromatic RingCount
1	2	1	18	20	6	6	1	2	0	1
2	2	1	22	24	6	6	1	2	0	1
3	2	1	25	27	6	6	1	2	0	1
4	2	1	25	27	6	6	1	2	0	1
5	2	2	23	26	12	12	2	2	0	2
6	2	1	26	28	6	6	1	2	0	1
7	2	1	24	26	6	6	1	2	0	1
8	2	1	24	26	6	6	1	2	0	1
9	2	2	24	27	12	12	2	2	0	2
10	2	1	22	24	6	6	0	3	1	0
11	2	1	22	24	6	6	1	2	0	1
12	1	1	16	17	6	6	1	1	0	1
13	1	2	17	19	12	12	2	1	0	2
14	1	1	18	19	6	6	1	1	0	1
15	1	1	19	20	6	6	1	1	0	1
16	1	1	20	21	6	6	1	1	0	1
17	1	1	21	22	6	6	1	1	0	1
18	1	1	20	21	6	6	1	1	0	1
19	1	1	20	21	6	6	1	1	0	1
20	2	1	18	20	6	6	1	2	0	1
21	2	1	19	21	6	6	1	2	0	1
22	2	1	26	28	6	6	1	2	0	1
23	2	2	20	23	11	11	1	3	1	1

24	2	1	19	21	5	5	0	3	1	0
25	2	1	19	21	5	5	0	3	1	0
26	2	1	23	25	5	5	0	3	1	0
27	2	1	23	25	5	5	0	3	1	0
28	2	1	23	25	5	5	0	3	1	0
29	2	0	20	21	0	0	0	2	0	0
30	2	0	20	21	0	0	0	2	0	0
31	2	0	23	24	0	0	0	2	0	0
32	2	0	23	24	0	0	0	2	0	0
33	2	0	21	22	0	0	0	2	0	0
34	2	0	21	22	0	0	0	2	0	0
35	2	0	26	27	0	0	0	2	0	0
36	2	0	27	28	0	0	0	2	0	0
37	2	1	21	23	6	6	0	3	1	0
38	1	1	16	17	6	6	1	1	0	1
39	2	0	21	22	0	0	1	1	0	0
40	2	1	24	26	5	5	0	3	1	0
41	2	1	25	27	5	5	0	3	1	0
42	2	1	25	27	5	5	0	3	1	0
43	2	1	25	27	5	5	0	3	1	0
44	2	1	20	22	6	6	1	2	0	1
45	2	1	21	23	6	6	1	2	0	1
46	2	1	21	23	5	5	0	3	1	0
47	2	0	25	26	0	0	0	2	0	0
48	2	0	23	24	0	0	0	2	0	0

No	FusedRing Count	LargestRing Size	Platt Index	Randic Index	Balaban Index	Harary Index	HyperWiener Index	Szeged Index	Wiener Index
1	2	6	72	11.59656	1.376373	84.89673	5731	2036	1439
2	2	6	84	13.4771	1.414243	105.9306	9922	3234	2221
3	2	6	92	14.94262	1.5986	124.9034	12157	3946	2759
4	2	6	94	14.8153	1.615176	125.845	11951	3918	2731
5	2	6	106	16.96026	1.342055	150.1276	16947	5440	3733
6	2	6	100	15.11594	1.61379	133.0712	12692	4159	2914
7	2	6	94	14.16725	1.669648	120.6034	10551	3516	2479
8	2	6	94	14.16725	1.691007	121.0634	10177	3408	2443
9	0	6	110	17.2781	1.16909	151.6126	23689	6310	4475
10	0	6	82	13.52841	1.308822	103.0614	11694	3350	2393
11	0	6	82	13.50724	1.325707	103.5631	11238	3190	2353
12	0	6	60	10.61339	1.542665	71.52252	4931	1428	1218
13	0	6	82	14.07972	1.376102	109.508	10610	2944	2423
14	0	6	66	11.56208	1.725573	82.42375	5983	1711	1479
15	0	6	70	11.93476	1.705671	88.12259	6583	1866	1623
16	0	6	76	12.2354	1.904295	94.32144	7186	2023	1769
17	0	6	74	12.99393	1.600249	94.9338	11011	2688	2249
18	0	6	70	12.54524	1.785426	91.29594	8126	2135	1881
19	0	6	72	12.41792	1.807371	92.07246	7953	2112	1858
20	2	6	72	11.59656	1.317596	83.66579	6221	1785	1497
21	2	6	74	12.09656	1.266742	88.60496	7227	2312	1683
22	2	6	96	15.3153	1.504224	129.7404	14548	4347	3105
23	2	6	96	14.95673	1.066024	120.5429	16294	4261	3146

24	2	6	72	11.59656	1.314469	83.66579	6221	1875	1497
25	2	6	72	11.59656	1.314469	83.66579	6221	1875	1497
26	2	6	88	13.21856	1.352999	105.0167	10830	2992	2310
27	2	6	88	13.20173	1.347966	104.6334	10872	2932	2318
28	2	6	88	13.20173	1.347966	104.6334	10872	2932	2318
29	0	5	56	9.630229	1.738991	63.22587	3268	969	899
30	0	5	56	9.630229	1.738991	63.22587	3268	969	899
31	0	5	64	11.07892	1.774064	77.36134	5309	1411	1329
32	0	5	64	11.07892	1.774064	77.36134	5309	1411	1329
33	0	6	58	10.13023	1.655952	67.71367	3927	1236	1037
34	0	6	58	10.13023	1.655952	67.71367	3927	1236	1037
35	0	6	72	12.47276	1.702687	90.76829	9320	2463	1984
36	0	6	80	12.64608	1.673299	97.03593	10292	2704	2169
37	0	6	78	13.09656	1.321536	97.13887	10509	2940	2189
38	0	6	60	10.61339	1.533402	71.43125	4971	1433	1223
39	0	5	58	10.11339	1.608776	66.9491	4161	1140	1066
40	2	6	92	13.62925	1.36016	111.7397	11426	3184	2466
41	2	6	94	14.16725	1.546827	117.8073	12207	3405	2651
42	2	6	98	13.95254	1.564759	118.9627	12025	3378	2624
43	2	6	98	13.93571	1.559873	118.5794	12067	3310	2632
44	2	6	82	12.33054	1.564451	96.7896	6996	2049	1733
45	2	6	84	12.83054	1.503231	101.951	8092	2630	1937
46	2	6	82	12.33054	1.561062	96.7896	6996	2151	1733
47	0	5	74	11.8129	1.813657	90.28277	5998	1637	1547
48	0	5	70	10.77489	1.965387	80.62709	4359	1291	1209

No	Asymmetric AtomCount	Rotatable BondCount	ChiralCenter Count	HeteroAliphatic RingCount	LargestRing SystemSize	RingSystem Count	Wiener Polarity	PolarSurface Area	HMOTotal PiEnergy	Surface Area
1	2	3	2	2	2	2	37	90.43	26.44856	503.4648
2	2	5	2	2	2	2	46	108.89	35.06515	631.6886
3	3	7	3	2	2	2	53	129.12	39.24515	672.8907
4	3	6	3	2	2	2	54	108.89	35.06515	648.3888
5	3	7	3	2	2	3	59	108.89	43.06515	719.249
6	3	6	3	2	2	2	56	108.89	35.06515	684.8888
7	3	5	3	2	2	2	51	99.66	30.75893	619.2829
8	3	5	3	2	2	2	51	99.66	30.75893	609.4926
9	3	6	3	2	1	4	59	93.67	51.12473	672.3752
10	2	4	2	2	1	3	43	130.35	33.20057	569.1745
11	2	4	2	2	1	3	43	110.74	33.39361	572.3828
12	2	5	2	1	1	2	29	99.22	26.44856	490.455
13	3	7	3	1	1	3	41	99.22	34.44856	604.8557
14	3	6	3	1	1	2	34	99.22	26.44856	546.7827
15	3	6	3	1	1	2	36	99.22	26.44856	555.5849
16	3	6	3	1	1	2	38	99.22	26.44856	558.7292
17	2	8	2	1	1	2	39	117.68	35.06515	621.4598
18	3	8	3	1	1	2	35	108.45	30.62856	594.1893
19	3	7	3	1	1	2	36	99.22	26.44856	542.7446
20	2	4	2	2	2	2	35	92.05	26.44856	520.5045
21	2	4	2	2	2	2	38	92.05	26.44856	538.6791
22	3	7	3	2	2	2	55	110.51	35.06515	710.4101
23	2	5	2	2	2	3	48	104.94	40.40966	652.3975

24	2	4	2	2	2	2	35	92.05	25.83841	519.9194
25	2	4	2	2	2	2	35	122.76	28.02551	492.6977
26	2	5	2	2	2	2	44	122.76	44.28551	542.8788
27	2	5	2	2	2	2	43	122.76	44.29173	575.3919
28	2	5	2	2	2	2	43	109.87	43.5807	577.8689
29	2	4	2	2	1	2	26	92.05	18.44856	473.9396
30	2	4	2	2	1	2	26	92.05	20.66856	466.5901
31	3	6	3	2	1	2	32	101.28	22.62856	553.6447
32	3	6	3	2	1	2	32	101.28	22.62856	560.4332
33	2	4	2	2	1	2	29	92.05	18.44856	480.2541
34	2	4	2	2	1	2	29	101.28	22.62856	479.8603
35	2	6	2	2	1	2	38	121.59	29.37056	620.8617
36	2	5	2	2	1	2	41	112.36	41.25645	545.9451
37	2	5	2	2	1	3	40	109.43	30.12741	577.1074
38	2	5	2	1	1	2	29	100.84	26.73509	490.8818
39	2	5	2	1	1	2	26	100.84	18.44856	479.7989
40	3	5	3	2	2	2	47	122.76	44.28551	554.0908
41	3	6	3	2	2	2	49	122.76	44.28551	574.6883
42	2	5	2	2	2	2	50	122.76	44.28551	566.4758
43	2	5	2	2	2	2	49	122.76	44.29173	596.0281
44	2	4	2	2	2	2	41	92.05	26.44856	545.1382
45	2	4	2	2	2	2	44	92.05	26.44856	565.481
46	2	4	2	2	2	2	41	92.05	25.83841	539.3937
47	3	6	3	2	1	2	38	101.28	22.62856	527.4219
48	3	4	3	2	1	2	35	92.05	18.44856	457.9401

No	WaterAccessible SurfaceArea	WaterAccessible SurfaceAreaHydrophobic	WaterAccessible SurfaceAreaPolar	WaterAccessible SurfaceAreaPlus	WaterAccessible SurfaceAreaNegative
1	503.4648	445.2253	58.23954	356.3596	147.1052
2	631.6886	545.0512	86.63739	491.8507	139.8379
3	672.8907	557.8455	115.0452	526.775	146.1157
4	648.3888	572.9874	75.40134	511.478	136.9108
5	719.249	647.6216	71.62734	559.5984	159.6506
6	684.8888	603.1741	81.71466	534.3102	150.5785
7	619.2829	556.4867	62.79613	477.1741	142.1088
8	609.4926	547.9908	61.50173	468.0056	141.4869
9	672.3752	549.1422	123.233	434.375	238.0001
10	569.1745	464.2385	104.936	399.6976	169.4769
11	572.3828	495.4279	76.9549	403.1683	169.2145
12	490.455	412.7942	77.66077	325.7016	164.7534
13	604.8557	536.5693	68.2864	411.2217	193.634
14	546.7827	474.7384	72.04428	373.0298	173.7529
15	555.5849	484.0218	71.56309	385.6802	169.9046
16	558.7292	488.7481	69.98111	389.5034	169.2258
17	621.4598	533.3295	88.13026	484.9663	136.4935
18	594.1893	512.5081	81.68116	436.4876	157.7017
19	542.7446	492.0856	50.65898	392.5176	150.2271
20	520.5045	432.0097	88.49484	374.3341	146.1704
21	538.6791	451.3556	87.32342	391.985	146.6941
22	710.4101	602.3147	108.0954	566.3535	144.0566
23	652.3975	505.4559	146.9417	448.5972	203.8003

24	519.9194	417.5578	102.3616	390.4804	129.439
25	492.6977	372.76	119.9377	368.6585	124.0393
26	542.8788	332.2062	210.6726	341.6681	201.2107
27	575.3919	335.9703	239.4215	357.8159	217.5759
28	577.8689	366.9588	210.9101	378.87	198.9989
29	473.9396	380.6281	93.31152	358.0442	115.8954
30	466.5901	362.6036	103.9865	363.8495	102.7406
31	553.6447	454.1628	99.48192	439.19	114.4548
32	560.4332	460.6236	99.80957	444.108	116.3251
33	480.2541	393.8001	86.45406	368.4736	111.7806
34	479.8603	377.7115	102.1489	380.048	99.81236
35	620.8617	502.0477	118.814	470.3463	150.5154
36	545.9451	342.7346	203.2105	353.1044	192.8407
37	577.1074	471.6091	105.4984	476.693	100.4145
38	490.8818	390.7849	100.0969	327.888	162.9937
39	479.7989	384.1522	95.64672	354.6582	125.1408
40	554.0908	354.1198	199.971	350.409	203.6819
41	574.6883	384.366	190.3223	371.6396	203.0487
42	566.4758	381.0632	185.4125	361.9311	204.5447
43	596.0281	381.7987	214.2294	376.2089	219.8192
44	545.1382	480.2899	64.84825	394.7736	150.3645
45	565.481	501.7074	63.77367	414.9922	150.4888
46	539.3937	455.4344	83.95931	406.0136	133.38
47	527.4219	457.3915	70.03038	427.4399	99.982
48	457.9401	399.4018	58.53826	356.6186	101.3214
